# Entecavir Interacts with Influx Transporters hOAT1, hCNT2, hCNT3, but Not with hOCT2: The Potential for Renal Transporter-Mediated Cytotoxicity and Drug–Drug Interactions

**DOI:** 10.3389/fphar.2015.00304

**Published:** 2016-01-05

**Authors:** Jana Mandíková, Marie Volková, Petr Pávek, Lucie Navrátilová, Lucie Hyršová, Zlatko Janeba, Jan Pavlík, Pavel Bárta, František Trejtnar

**Affiliations:** ^1^Department of Pharmacology and Toxicology, Faculty of Pharmacy in Hradec Králové, Charles University in PragueHradec Králové, Czech Republic; ^2^Institute of Organic Chemistry and Biochemistry, Academy of Sciences of the Czech RepublicPrague, Czech Republic; ^3^Cayman Pharma Ltd.Neratovice, Czech Republic

**Keywords:** antivirals, nephrotoxicity, renal disposition, drug–drug interactions

## Abstract

Entecavir (ETV) is one of the most potent agents for the treatment of the hepatitis B viral infection. The drug is principally eliminated by the kidney. The goal of this study was to investigate the potential of ETV to interact *in vitro* with the renal SLC transporters hOAT1, hOCT2, hCNT2 and hCNT3. Potential drug–drug interactions of ETV at the renal transporters with antiviral drugs known to be excreted by the kidney (adefovir, tenofovir, cidofovir) as well as transporter-dependent cytotoxicity were also examined. Interactions with the selected transporters along with cytotoxicity were studied in several transiently transfected cellular models using specific substrates and inhibitors. ETV was found to be both a substrate and inhibitor of hOAT1 (IC_50_ = 175.3 μM), hCNT2 (IC_50_ = 241.9 μM) and hCNT3 (IC_50_ = 278.4 μM) transporters, although it interacted with the transporters with relatively low affinities. ETV inhibited the cellular uptake of adefovir, tenofovir, and cidofovir by hOAT1; however, effective inhibition was shown at ETV concentrations exceeding therapeutic levels. In comparison with adefovir, tenofovir, and cidofovir, ETV displayed no transporter-mediated cytotoxicity in cells transfected with hOAT1, hCNT2, and hCNT3. No significant interaction of ETV with hOCT2 was detected. The study demonstrates interactions of ETV with several human renal transporters. For the first time, an interaction of ETV with the hCNTs was proved. We show that the potency of ETV to cause nephrotoxicity and/or clinically significant drug-drug interactions related to the tested transporters is considerably lower than that of adefovir, tenofovir, and cidofovir.

## Introduction

Entecavir (ETV), a synthetic guanosine analog, is one of the most potent and highly selective agents for the treatment of chronic hepatitis B (HBV) infection. The active 5′-triphosphate of ETV inhibits the replication of HBV at all three steps of the synthesis process (Scott and Keating, 2009). ETV is eliminated primarily in the urine by glomerular filtration and tubular secretion (Matthews, 2006; Razonable, 2011). For various purposes ETV may be combined in therapy with various antivirals or other drugs. Recently, combinations of ETV and other anti-HBV drugs such as adefovir or tenofovir have been demonstrated to be effective in patients with some types of resistance to antiviral therapy (Sheng et al., 2011; Chae et al., 2012).

The solute carrier family (SLC) of membrane transporters located in the renal proximal tubular cells have been shown to interact with numerous widely used antivirals (Cihlar et al., 2009; Minuesa et al., 2011). Transport systems for organic anions (OATs) and cations (OCTs) have been found to transport a variety of compounds in kidney cells (Koepsell and Endou, 2004; El-Sheikh et al., 2008). The most abundantly expressed types of OATs and OCTs in human renal tubular cells are human organic anion transporter 1 (hOAT1) and human organic cation transporter 2 (hOCT2), respectively (Klaassen and Aleksunes, 2010). Several guanosine antivirals show substrate specificity to the OAT and OCT families (Minuesa et al., 2011). For example, antiviral agents from the group of acyclic nucleoside phosphonates such as adefovir, tenofovir, and cidofovir have been characterized as substrates of hOATs (Uwai et al., 2007).

The possible involvement of OATs and OCTs in the renal excretion of ETV has been suggested in rats *in vivo* (Yanxiao et al., 2011). Recently, OAT1, OAT3 and OCTs have been described to transport ETV in genetically engineered cell lines and kidney slices (Xu et al., 2013).

Concentrative nucleoside transporters (SLC28A1-3; CNTs) may also play an important role in the transmembrane transport of synthetic nucleoside analogs into various cells (Gray et al., 2004; Errasti-Murugarren et al., 2010). CNTs mediate the unidirectional uptake of nucleosides in an active process (Molina-Arcas and Pastor-Anglada, 2010). The types 1 (hCNT1), 2 (hCNT2), and 3 (hCNT3) are typically expressed in the proximal tubular cells of human kidney (Mangravite et al., 2003; Klaassen and Aleksunes, 2010). CNT1 and CNT2 are pyrimidine nucleoside-preferring and purine nucleoside-preferring carriers, respectively, whereas CNT3 shows broader substrate selectivity (Gray et al., 2004). Interestingly, ribavirin, a guanosine analog antiviral agent, has been detected as a high-affinity substrate of hCNT2 and hCNT3 (Yamamoto et al., 2006; Cano-Soldado and Pastor-Anglada, 2012). However, interaction of ETV with hCNT2 and hCNT3 has not been studied so far.

In the current study we hypothesized that human renal drug influx transporters, including hOAT1, hOCT2, hCNT2 and hCNT3, might be involved in the handling of ETV in the renal tubules. These transporters could be potential sites for the interaction of ETV with other antiviral drugs such as tenofovir, adefovir, and cidofovir transported by the same renal transport systems. Importantly, interactions with the selected transporters could result in nephrotoxicity caused by transport-related accumulation.

Along these lines of inquiry, the aim of this study was to investigate the interactions of ETV with the selected renal SLC transporters *in vitro* using genetically engineered cell models. We also analyzed potential drug-drug interactions of ETV with adefovir, tenofovir, and cidofovir. Finally, we compared the cytotoxicity of the tested antivirals and assessed the significance of OAT1 transporter for *in vitro* toxic effects of ETV and comparators.

## Materials and Methods

### Chemical Reagents Used

Entecavir, adefovir, and tenofovir were obtained from Santa Cruz Biotechnology (Paso Robles, CA, USA). Cidofovir was purchased from Sigma Aldrich (St. Louis, MO, USA). [Adenine-2,8-^3^H]-adefovir ([^3^H]adefovir), [adenine-2,8-^3^H]-tenofovir ([^3^H]tenofovir), [5-^3^H]-cidofovir ([^3^H]cidofovir) and [5-^3^H]-uridine ([^3^H]uridine) were obtained from Moravek Biochemicals (Brea, CA, USA). The *p*-[glycyl-2-^3^H]-aminohippuric acid ([^3^H]PAH) was purchased from Perkin Elmer (Waltham, MA, USA). Radiolabeled ETV ([^3^H]ETV) and methyl-4-phenylpyridinium acetate ([^3^H]MPP+) were obtained from American Radiolabeled Chemicals (St. Louis, MO, USA). Expression plasmids were obtained from OriGene Technologies (Rockville, MD, USA).

### Cell Culturing

The human cervical epithelioid carcinoma cell line (HeLa) and Madin–Darby canine kidney cell line (MDCK II) were purchased from the European Collection of Cell Culture (Salisbury, UK). The HeLa cells were routinely cultured in Eagles minimum essential medium (EMEM). The MDCKII were routinely cultured in Dulbecco’s modified Eagle’s medium (DMEM).

### Transfection

The HeLa cells were seeded at a density of 7 × 10^4^ cells per well in 24-well plates. The MDCK II cells were seeded at a density of 2 × 10^5^ cells per well in 24-well plates. In cytotoxicity assays, the HeLa cells were seeded at a density of 15 × 10^3^ cells per well. The following day the cells were transiently transfected with appropriate plasmid coding for the studied transporter or empty vector pCMV6-Entry (pCMV6, empty vector) using the Lipofectamine 2000 reagent (Invitrogen, Carlsbad, CA, USA) and Opti-MEM (Invitrogen, Carlsbad, CA, USA) according to the manufacturer’s protocol. The cells transiently transfected with the appropriate empty vector served as a control. The overexpression of the studied transporters was checked by Western blot analysis and by function tests using an accumulation study with tritium-labeled prototypical substrates.

### Interactions with SLC Transporters

Inhibitory transport assays were carried out in 24-well plates as described previously (Mandikova et al., 2013). Transport assays in HeLa cells transiently transfected with the hOAT1 expression construct were performed 48 h after transfection. Transport assays in MDCK II cells transiently transfected with hOCT2, hCNT2 or hCNT3 were performed 24 h after transfection. The cultivation medium was removed and the cells were washed with transport solution and preincubated for 10 min at 37°C. The standard radiolabeled substrate or tested radioactive antiviral substance dissolved in transport solution was added to the cells and incubated for 2 min. [^3^H]PAH (1 μM) was used as a prototypical substrate for OATs, [^3^H]MPP^+^ (1 μM) for OCTs and [^3^H]uridine (1 μM) for CNTs. The rate of inhibition of intracellular accumulation of the radioactive substrates induced by gradually increasing concentrations of ETV (0–2000 μM) was used as a measure of the inhibitory effect on the transporter. [^3^H]adefovir (0.1 μM), [^3^H]tenofovir (0.5 μM) and [^3^H]cidofovir (70 μM) were used in the interaction experiments with ETV. After the designated time period, the incubation was stopped by washing the cells twice with ice-cold solution containing 137 mM NaCl and 10 mM HEPES, pH 7.4. The cells were disintegrated with 0.1 mL of Triton X 0.5% in 100 mM NaOH for 1 h.

To test if the ETV was a substrate of the tested influx transporters, the accumulation of [^3^H]ETV was studied in hOAT1, hOCT2, hCNT2 and hCNT3 transfected cells during a 5 min period. To determine the kinetic parameters, *K*_m_ and *V*_max_, [^3^H]ETV in increasing concentrations (0–500 μM) in triplicates was incubated for 5 min with the cells transfected by hOAT1, hCNT2 or hCNT3.

The results regarding inhibition were expressed as the concentration of inhibitor IC_50_, which resulted in the half-rate inhibition of transport of the labeled substrate. IC_50_ values were calculated using non-linear regression analysis using GraphPad Prism software (version 6). The kinetic parameters *K*_m_ and *V*_max_ for the uptake of ETV was derived from a non-linear regression analysis of the Michaelis–Menten model using GraphPad Prism software (version 6).

### Cytotoxicity Assays

The Hela cells were plated in a 96-well plate and transfected with hOAT1 as described above. After transfection, the cells were treated with ETV, adefovir, tenofovir, or cidofovir at concentrations 50–1000 μM in triplicates. To determine potential ETV cytotoxicity mediated by hCNT2 or hCNT3, the MDCK II cells were plated in a 96-well plate, transfected with hCNT2 or hCNT3a, and treated with ETV at concentrations 250–1000 μM. The controls were prepared simultaneously. After 24 h of incubation, the reagent from the kit CellTiter 96 AQueous One Solution Cell Proliferation Assay (CellTiter 96, PROMEGA, USA) was added. After 1.5 h of incubation at 37°C the absorbance was recorded at 490 nm.

### Western Blot Analysis

The HeLa cells and the MDCK II cells were transiently transfected with the transporters of interest as described above. As negative control cells, transiently transfected with empty vector were used. After protein separation and transmission to polyvinylidene membrane (Sigma–Aldrich, St. Louis, MO, USA), the membrane was incubated with anti-SLC22A6 (AB1), anti-SLC28A2, anti-SLC28A3 (Sigma–Aldrich, St. Louis, MO, USA), anti-SLC22A2 (Abcam, Cambridge, UK) or anti-β-actin antibody (Sigma–Aldrich, St. Louis, MO, USA). β -actin was used as a loading control. Detection was performed with appropriate peroxidase-conjugated secondary antibodies (GE Healthcare, Little Chalfont, UK; Sigma–Aldrich, St. Louis, MO, USA). The immunoreactive bands on the X-ray film (FOMA Bohemia, Hradec Kralove, Czech Republic) were scanned with the calibrated CCD camera Image Quant 400 (GE Healthcare, Little Chalfont, UK).

### Statistical Analysis

Data obtained in the experiments are expressed as mean ± SD. All experiments were performed in triplicates. The statistical significance of the difference in the parameters was determined using a two-way ANOVA test (interaction with SLC transporters) in GraphPad Prism software (version 6). Differences were considered significant at *p*-value <0.05.

## Results

### Validation of the Transport Studies

**FIGURE 1:**
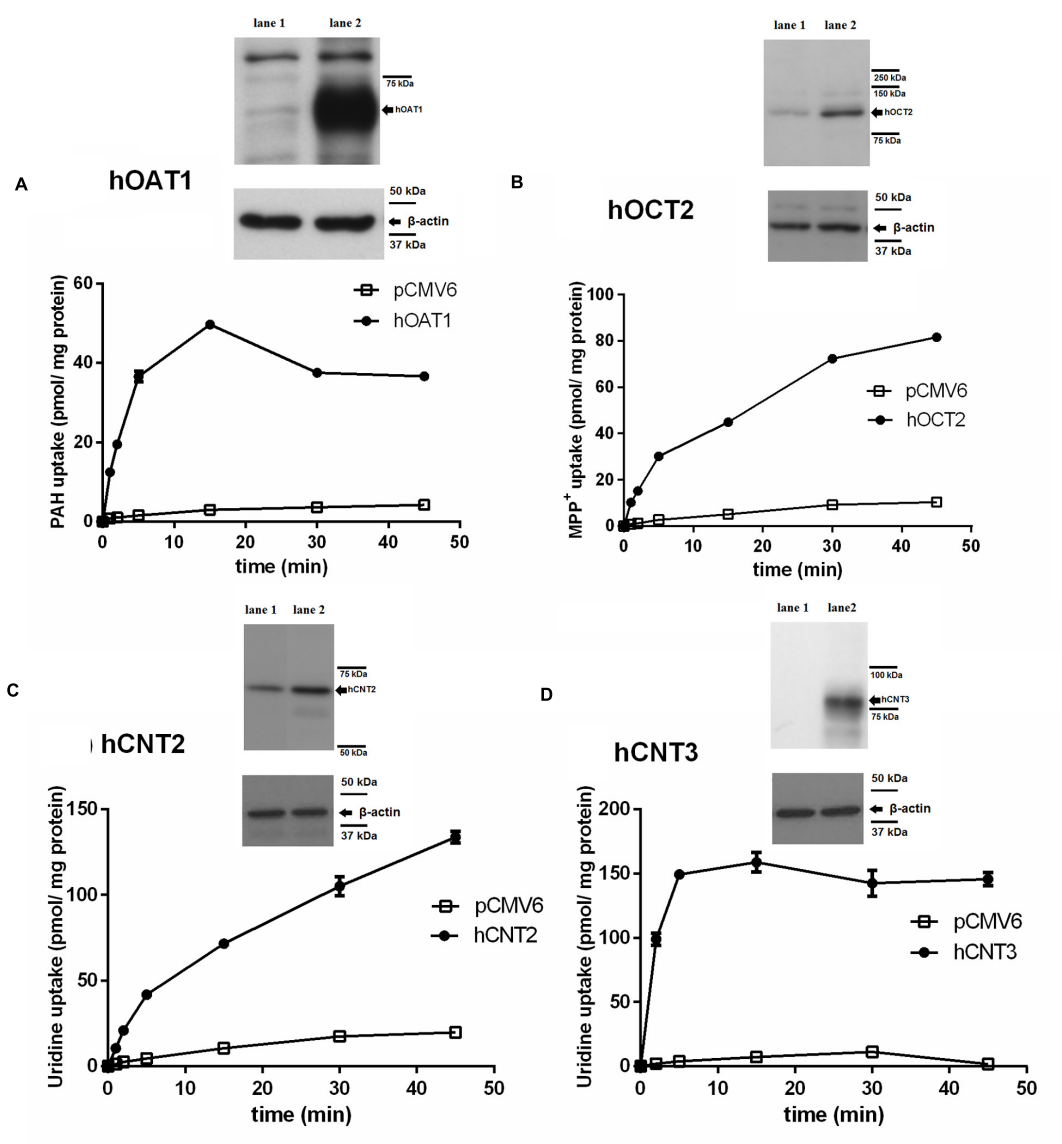
**Time-dependent uptake of typical substrates in transiently transfected cell models and demonstration of overexpression of transporters in transfected cells using Western blot analysis.** The time profile of the uptake of 1 μM [^3^H]PAH via hOAT1 (filled circles) and empty vector (pCMV6) transfected (open squares) HeLa cells **(A)**. The time profile of the uptake of 1 μM [^3^H]MPP^+^ via hOCT2 (filled circles) and empty vector transfected (open squares) MDCK II cells **(B)**. The time profile of the uptake of 1 μM [^3^H]uridine via hCNT2 (filled circles) and empty vector transfected (open squares) MDCK II cells **(C)**. The time profile of the uptake of 1 μM [^3^H]uridine via hCNT3 (filled circles) and empty vector transfected (open squares) MDCK II cells **(D)**. Experiments were performed in triplicates. Western blot analysis: overexpression of the appropriate transporter (lane 2) or negative control (empty vector transfected cells, lane 1) in cell lysates.

To check the validity of the transport studies, the time profile of the uptake of each of the [^3^H]-labeled standard substrates in the appropriate transfected cell line was determined (**Figure 1**). The intracellular accumulation of [^3^H]PAH (**Figure 1A**), [^3^H]MPP^+^ (**Figure 1B**) and [^3^H]uridine (**Figures 1C,D**) in the cells transfected with the appropriate transporter was linear within 5 min, and it was also markedly higher in the cells transfected with the transporter of interest than in the cells transfected with the empty vector. Using an interval of 2 min of incubation it was demonstrated that the uptake of all typical substrates tested in the appropriate cell model was at least 13-fold higher than in the control cells transfected with empty vector.

### Evaluation of Transfection Effectiveness by Western Blotting

To confirm the expression of the tested SLC transporters after transient transfection, a Western blot analysis of protein extracts from the cells transfected with studied transporters was performed. As the controls, HeLa and MDCK II cells transfected with the empty vector were used. A significant increase in the level of the tested transporters was demonstrated in comparison with the cells transfected with empty vector (**Figure 1**).

### Interactions of Entecavir and Comparators with SLC Transporters

#### Inhibitory Effect of ETV on Transporters

**FIGURE 2:**
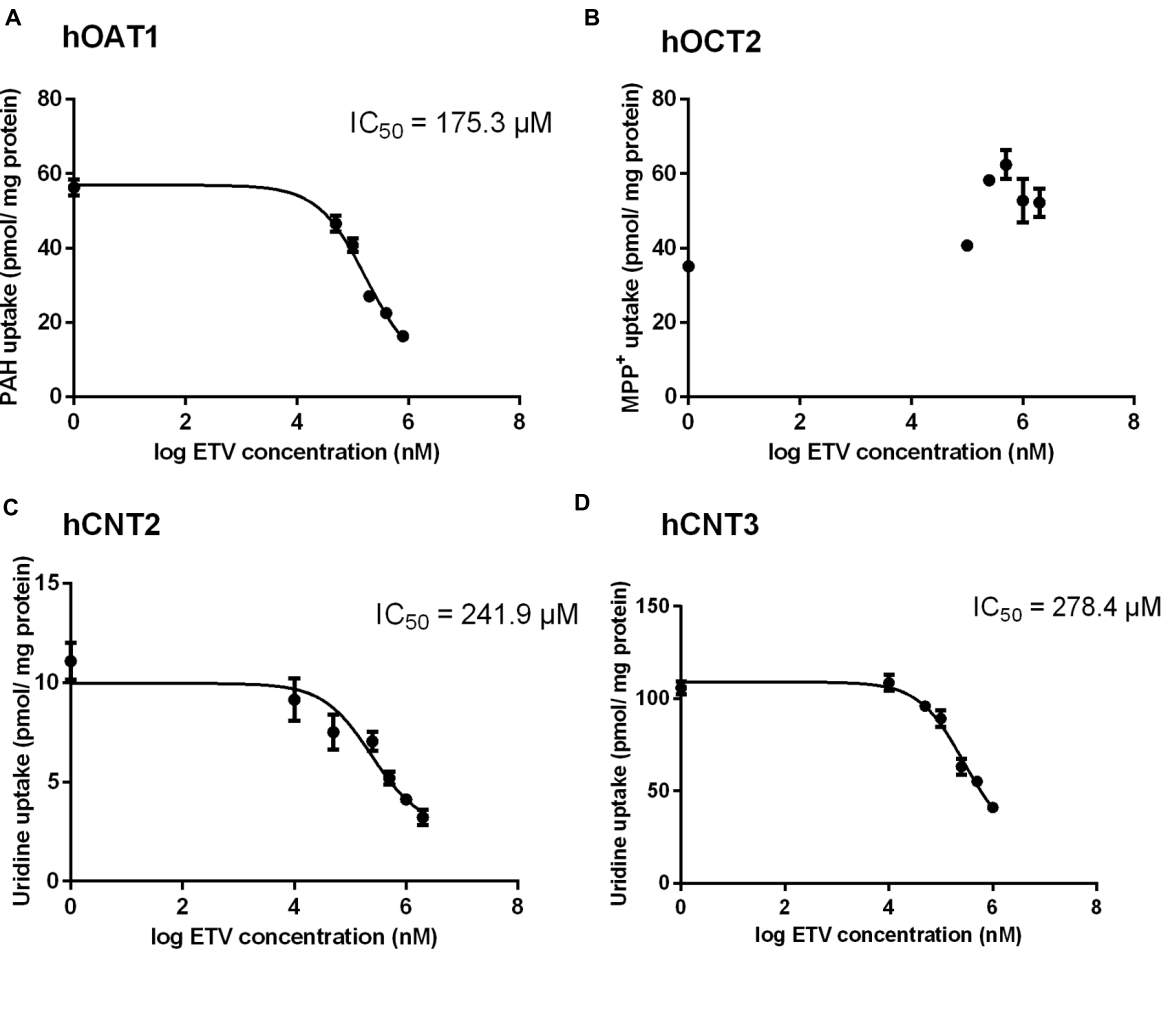
**Effect of ETV on the accumulation of typical substrates of the studied transporters.** The HeLa cells transiently transfected with the expressing vector for hOAT1 **(A)** and MDCK II cells transiently transfected with the expressing vector for hOCT2 **(B)**, hCNT2 **(C)**, hCNT3 **(D)** were incubated with 1 μM [^3^H]PAH **(A)**, 1 μM [^3^H]MPP^+^ **(B)** and 1 μM [^3^H]uridine **(C,D)** in the presence of gradually increasing concentrations of ETV (0–2000 μM) for 2 min at 37°C in triplicates. Each point represents the mean ± SD of the accumulated amounts of radiotracer. The IC_50_ value represents the inhibitory concentration of ETV calculated using non-linear regression analysis. The value of radiotracer accumulation in the cells transfected with empty vector was subtracted.

To prove whether ETV is an inhibitor of the tested SLC transporters, the cell line transiently transfected by hOAT1, hOCT2, hCNT2 or hCNT3 were incubated with [^3^H]-labeled typical substrates in combination with unlabeled ETV. An evaluation of the potential to inhibit accumulation of the standard substrates in the cell models revealed a considerable effect of ETV on hOAT1, hCNT2 and hCNT3 mediated transport, with IC_50_ being 175.3; 241.9, and 278.4 μM, respectively (**Figure 2**). No interaction of ETV with hOCT2 was observed under the used conditions (**Figure 2**).

#### ETV Influx by Transporters

**FIGURE 3:**
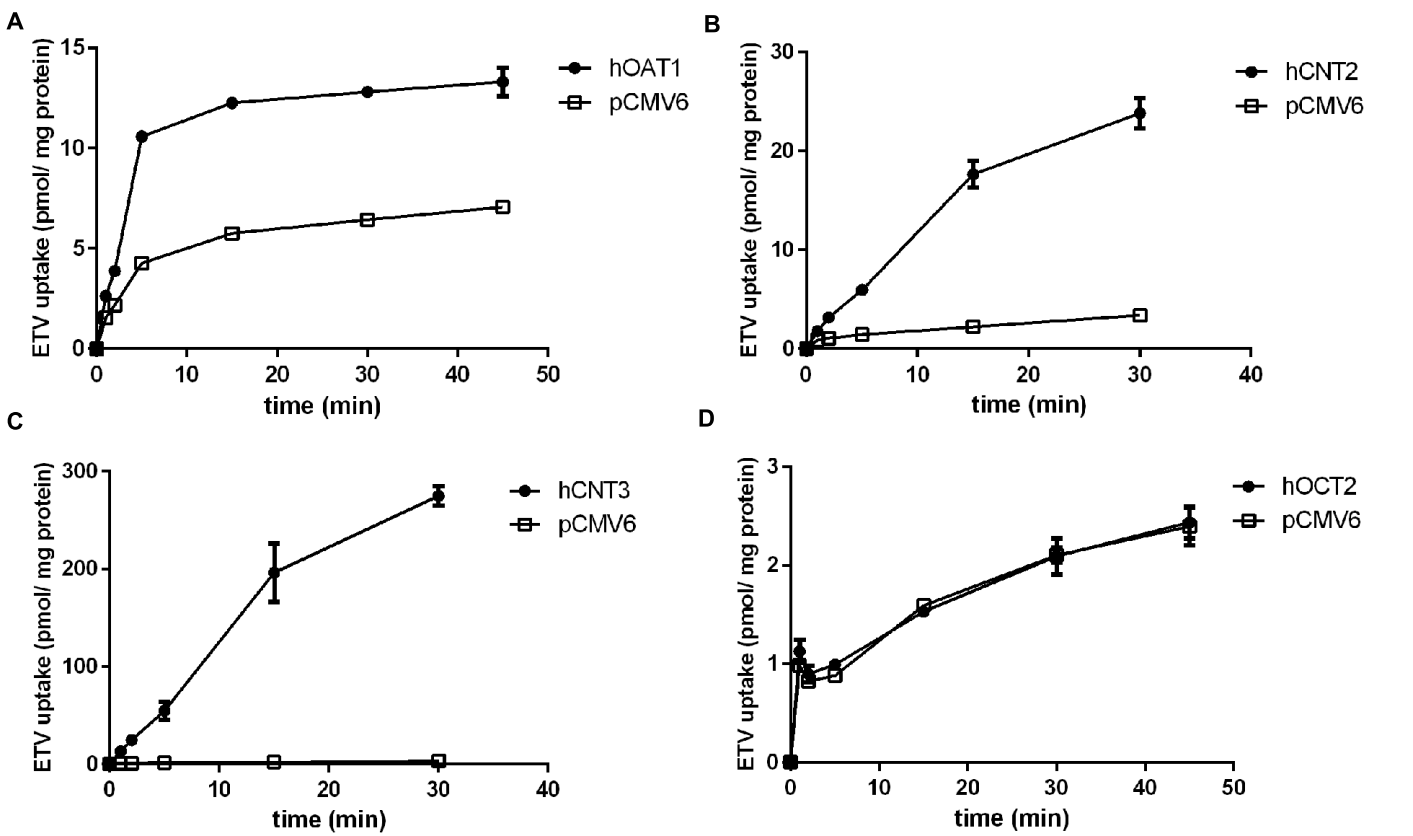
**The time-dependent profile of [^3^H]ETV uptake.** The time profile of the uptake of 0.1 μM [^3^H]ETV in hOAT1 **(A)**, hCNT2 **(B)**, hCNT3 **(C)** and hOCT2 **(D)** transiently transfected cells (filled circles) or empty vector (pCMV6) transfected cells (open squares) at 37°C in triplicates. The uptake was linear within first 5 min of incubation. Each point represents the mean ± SD of the accumulated amount of [^3^H]ETV.

**FIGURE 4:**
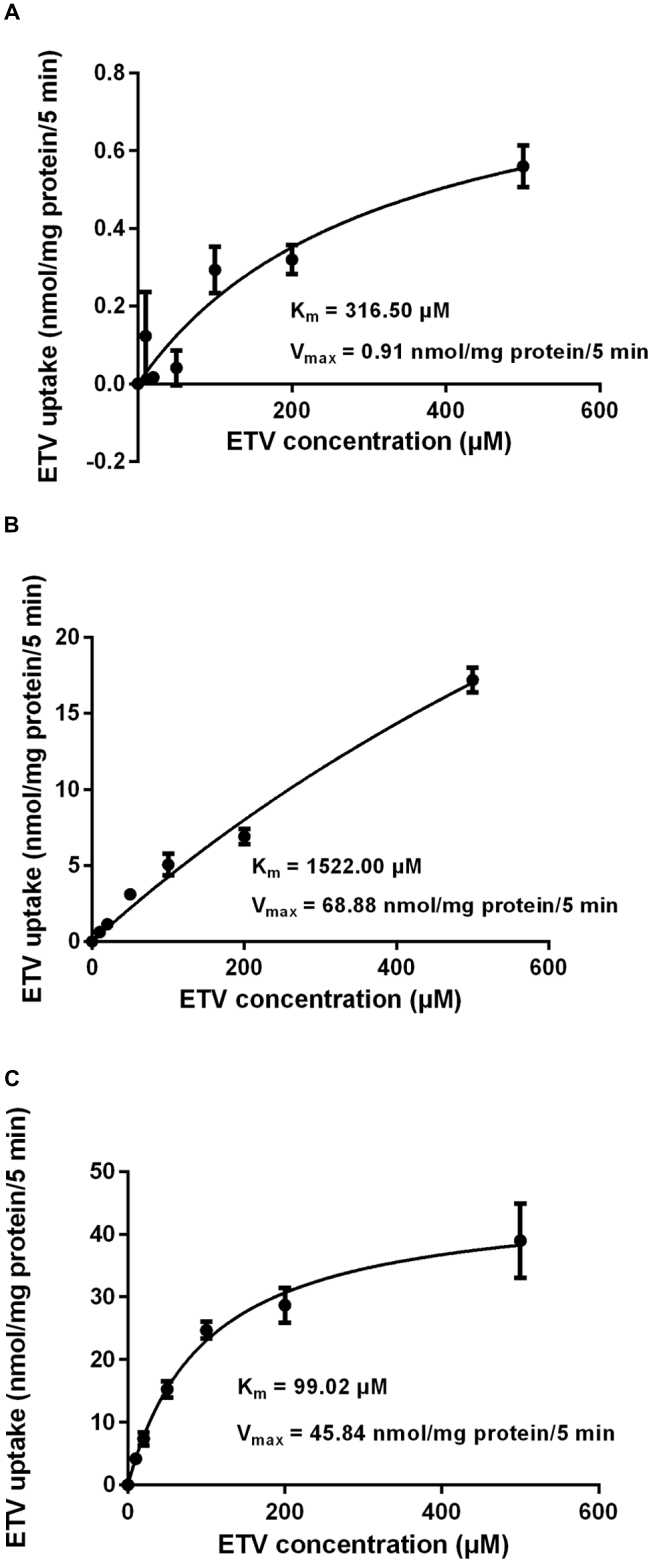
**Concentration-dependent uptake of [^3^H]ETV.** The uptake of [^3^H]ETV in hOAT1 **(A)**, hCNT2 **(B)**, and hCNT3 **(C)** transiently transfected cells was measured at different concentrations after 5 min of incubation at 37°C in triplicates. Each point represents the mean ± SD of the accumulated amount of [^3^H]ETV. The value of radiotracer accumulation in the cells transfected with empty vector was subtracted.

In order to determine whether ETV is a substrate of the tested transporters, a set of uptake transport studies with [^3^H]ETV in the cells transfected by hOAT1, hOCT2, hCNT2 and hCNT3 was carried out (**Figure 3**). In time dependent studies, the uptake of [^3^H]ETV was linear in the first 5 min. The uptake of [^3^H]ETV in the hOAT1, hCNT2 and hCNT3 transfected cells was significantly higher than in the empty vector (pCMV6) transfected control cells (**Figures 3A–C**). ETV was found to be a substrate of hOAT1, hCNT2 and hCNT3. The appropriate kinetic parameters are presented in **Figure 4**. The transport efficiency (*V*_max_/*K*_m_) values (μl/5 min/mg) for hOAT1, hCNT2 and hCNT3 were 2.88, 45.2 and 462.9, respectively. In contrast, we did not find any interaction of ETV with hOCT2 (**Figure 3D**).

#### Comparative Study and Interactions with Selected Antivirals

**FIGURE 5:**
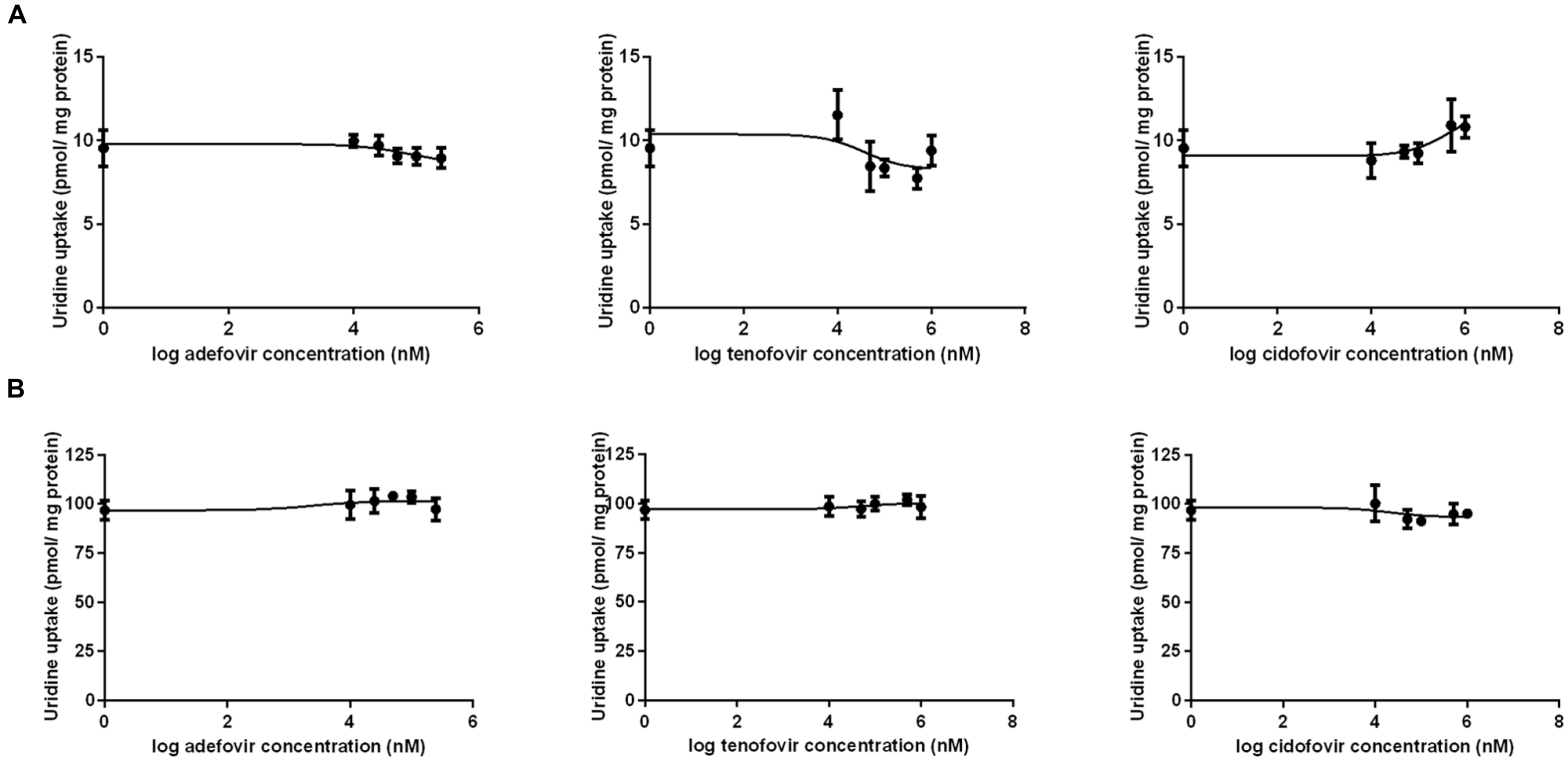
**Comparison of the inhibitory effect of adefovir, tenofovir, and cidofovir on hCNT2 and hCNT3.** The MDCK II cells transiently transfected with the expressing vector for hCNT2 **(A)** and hCNT3 **(B)** were incubated with 1 μM [^3^H]uridine in gradually increasing concentrations of adefovir, tenofovir, and cidofovir (0–1000 μM) for 2 min at 37°C in triplicates. Each point represents the mean ± SD of the accumulated amounts of 1 μM [^3^H]uridine. The value of radiotracer accumulation in the cells transfected with empty vector was subtracted.

**FIGURE 6:**
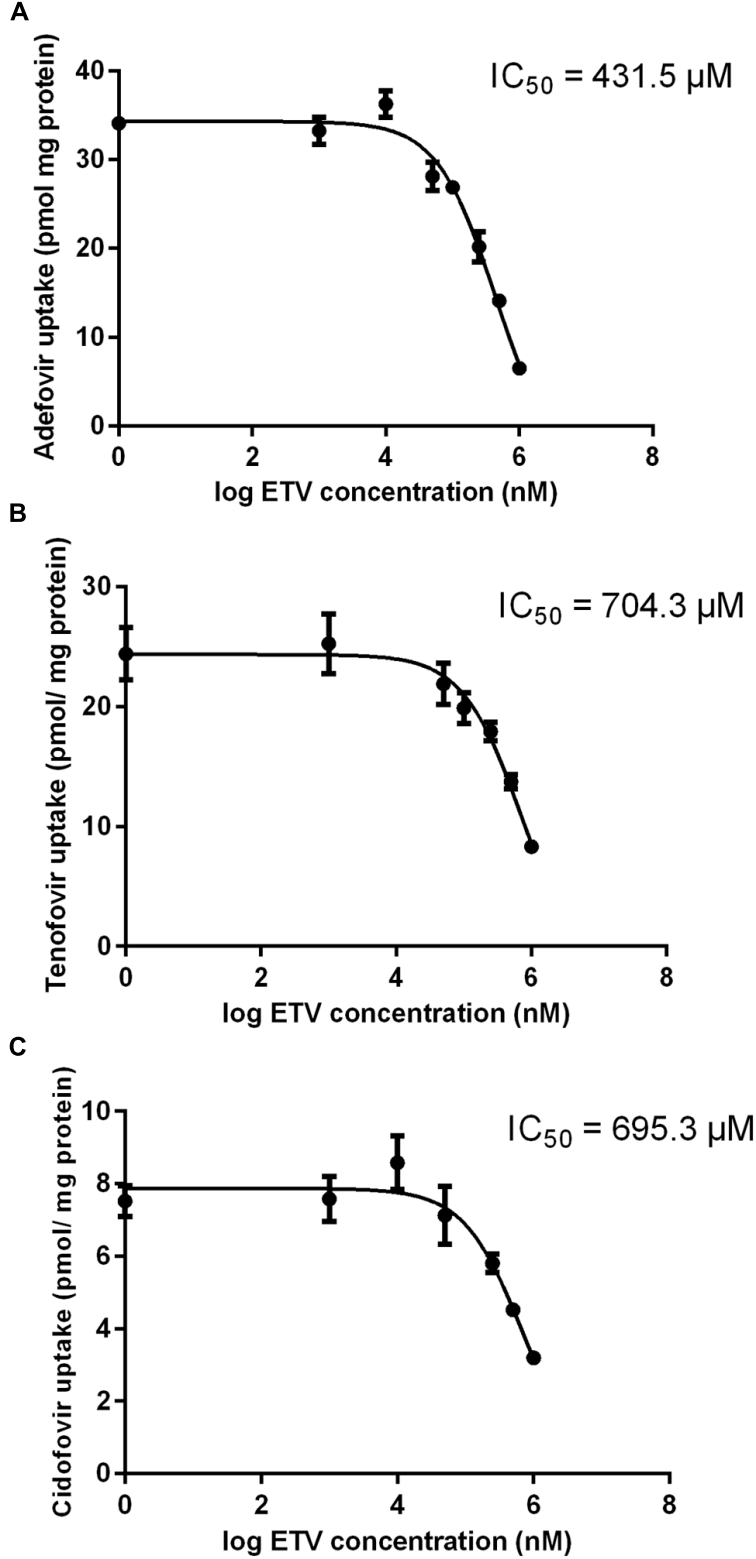
**Effect of ETV on adefovir, tenofovir, and cidofovir accumulation mediated by OAT1 in HeLa cells.** The accumulation of [^3^H]adefovir (0.1 μM) **(A)**, [^3^H]tenofovir (0.5 μM) **(B)** and [^3^H]cidofovir (70 μM) **(C)** was inhibited by ETV (0–1000 μM) for a 2 min period in HeLa cells transiently transfected with hOAT1. Each point represents the mean ± SD of the accumulated amounts of radiotracer. The IC_50_ value represents the inhibitory concentration of ETV calculated using non-linear regression analysis. The value of radiotracer accumulation in the cells transfected with empty vector was subtracted.

The results of a comparative study on the inhibitory effect of adefovir, tenofovir, and cidofovir on hCNT2 and hCNT3 are presented in **Figure 5**. Interactions of adefovir, tenofovir, and cidofovir with hOAT1 have been shown previously (Mandikova et al., 2013). Significant interactions of adefovir, tenofovir, and cidofovir with hCNT2 (**Figure 5A**) and hCNT3 (**Figure 5B**) were not observed. To evaluate the potential interactions of ETV with the other studied antivirals at hOAT1, the accumulation of [^3^H]-labeled adefovir, tenofovir, and cidofovir was studied in HeLa cells transfected with hOAT1 in combination with increasing concentrations of ETV. The results demonstrated a considerable inhibition of the uptake of all the tested antivirals by ETV in the model cells (**Figure 6**).

### Effect of OAT1, CNT2 and CNT3 on Cytotoxicity of Entecavir

**FIGURE 7:**
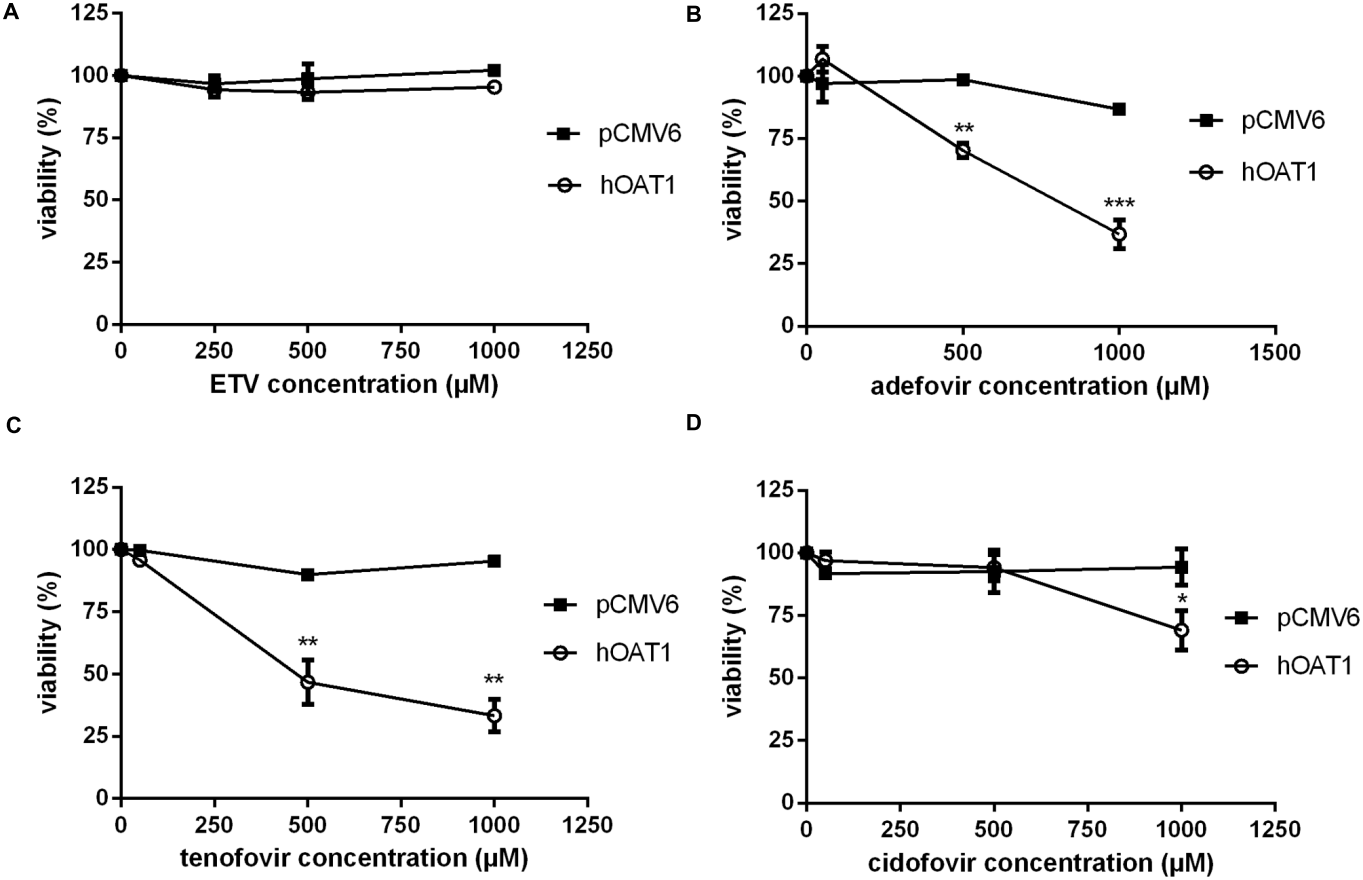
**Effect of hOAT1 expression on cytotoxicity of the tested antiviral drugs.** HeLa cells transiently transfected with hOAT1 (open circles) and empty vector pCMV6 (closed squares) were incubated for 24 h with three various concentrations of ETV **(A)**, adefovir **(B)**, tenofovir **(C)** and cidofovir **(D)**. The experiments were carried out simultaneously with hOAT1 and empty vector transfected cells in triplicates. Data are presented as means ± SD. The values from the end point were subjected to statistical analysis. ^∗^*p* < 0.05; ^∗∗^*p* < 0.01; ^∗∗∗^*p* < 0.001 compared with control.

**FIGURE 8:**
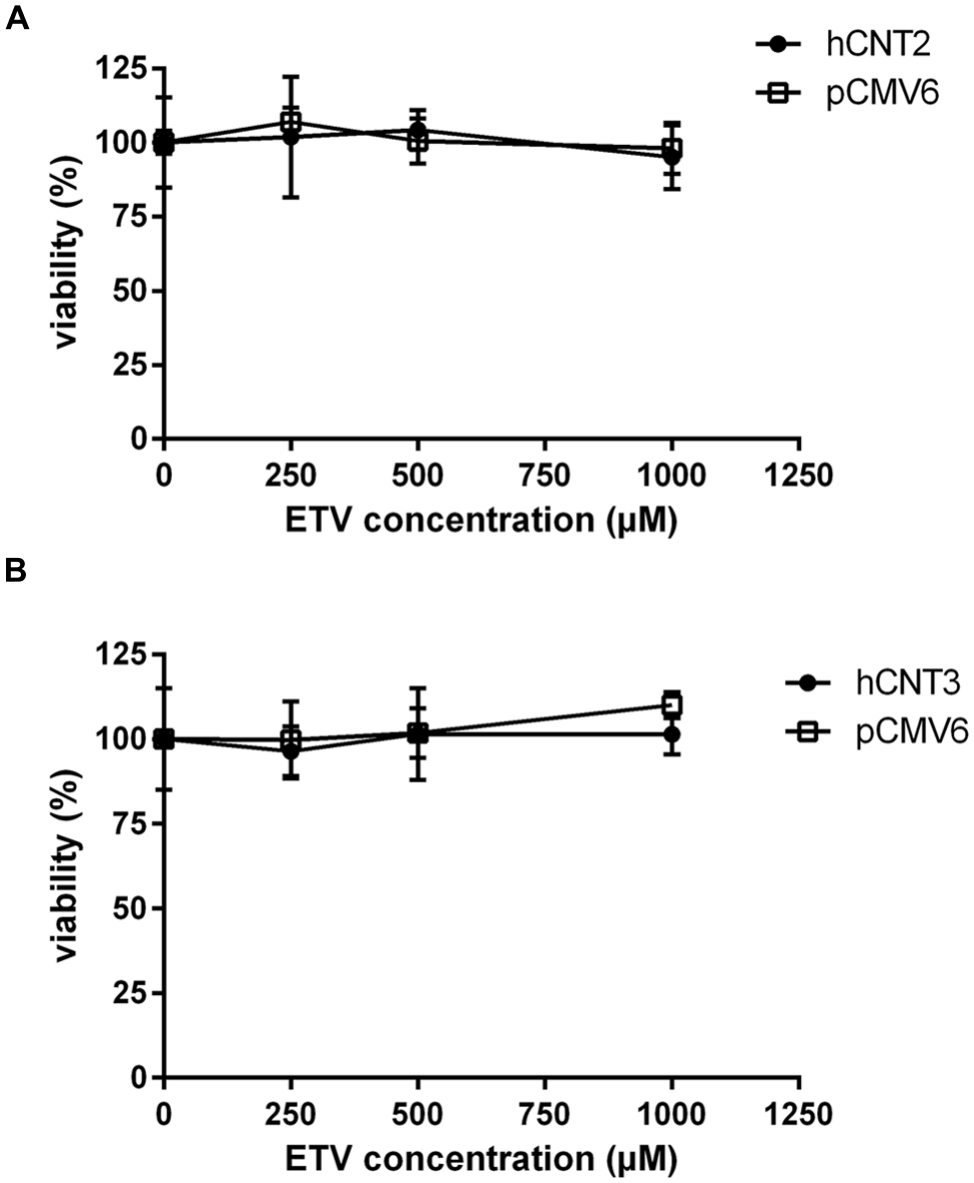
**Effect of hCNT2 and hCNT3 expression on cytotoxicity of ETV.** MDCK II cells transiently transfected with hCNT2 or CNT3 (closed circles) or empty vector pCMV6 (open squares) were incubated for 24 h with three various concentrations of ETV. The experiments were carried out simultaneously with hCNT2 **(A)** or hCNT3 **(B)** and empty vector transfected cells in triplicates. Data are presented as means ± SD. The values from the end point were subjected to statistical analysis. ^∗^*p* < 0.05; ^∗∗^*p* < 0.01; ^∗∗∗^*p* < 0.001 compared with control.

To measure the relation between the effect of the tested agents on cell viability and ability to interact with hOAT1, a standard MTS colorimetric cell viability assay was performed using hOAT1 transfected cells. As shown in **Figure 7A**, the treatment of ETV at the concentrations tested (250, 500, and 1000 μM) had no significant effect on cell viability in hOAT1 transfected cells in comparison with the control cells. The high expression of hOAT1 significantly enhanced the cytotoxic effect of adefovir, tenofovir, and cidofovir in comparison with the empty vector transfected cells (**Figures 7B–D**). The study in cells overexpressing hCNT2 or hCNT3 did not prove any significant increase in cytotoxicity in the transfected cells in comparison with the controls (**Figure 8**).

## Discussion

To study the role of the individual transporters contributing to ETV renal handling, we selected the main types of drug transporters abundantly expressed in human kidney which might be potentially involved in ETV transmembrane transport. This work builds on an *in vivo* study which suggested an involvement of organic anion and cation transporters in the renal excretion of ETV (Yanxiao et al., 2011). The authors of this study have demonstrated that both probenecid and cimetidine show an inhibitory effect on the renal excretion of ETV after intravenous coadministration. Probenecid is a commonly used potent inhibitor of OATs (Cihlar et al., 1999; Burckhardt and Burckhardt, 2003). Thus our results support the above-mentioned data found *in vivo* and confirm ETV interaction with the human renal influx transporter for organic anions hOAT1. The found data confirmed the findings by Xu et al. (2013) who demonstrated interactions of ETV with hOATs using a similar *in vitro* cellular model. The value of *K*_m_ = 316.5 μM found for hOAT1 is in accordance with the previously published value of 250 μM (Xu et al., 2013). In contrast to the findings in rats *in vivo* (Yanxiao et al., 2011) and in an *in vitro* study using rat renal slices (Xu et al., 2013) suggesting the participation of OCTs in the transport of ETV, we did not prove any interaction of ETV with hOCT2 in the used *in vitro* cell model (**Figure 3D**). A relevant explanation for these contradictory findings may be a possible contribution of other subtypes of OCTs to renal transport *in vivo* and in the rat renal slices. Since we employed the model of human OCT2, another explanation may be interspecies differences in ETV affinity to OCTs, as the other studies mentioned used rat experimental models.

Our experiments enabled the direct comparison of the ability of the tested antivirals to interact with hOAT1 in one experimental model. The investigation showed that the potential of ETV to inhibit the transport of [^3^H]PAH into transfected cells seems to be considerably lower than that of adefovir, tenofovir, and cidofovir (Mandikova et al., 2013). The tested antiviral agents can be arranged according to the inhibitory effect on hOAT1 in the following descending order: adefovir > tenofovir ≈ cidofovir >> ETV. Based on the proved potency of ETV to inhibit hOAT1, experiments focusing on the drug–drug interactions of ETV with three known substrates of hOATs, adefovir, tenofovir, and cidofovir (Cihlar et al., 1999; Uwai et al., 2007) were performed. We used concentrations of adefovir (0.1 μM), tenofovir (0.5 μM) and cidofovir (70 μM), which correspond to therapeutic plasma levels achieved in humans (Cundy, 1999; Kearney et al., 2005; Delahunty et al., 2006). We demonstrated the considerable potency of ETV to inhibit transport by hOAT1 in all three antiviral agents in high micromolar concentrations (**Figure 6**). Still, the therapeutic concentrations of ETV in plasma following regular doses have been declared to be at below 50 nM (Matthews, 2006; Yan et al., 2006). Therefore the clinical significance of these drug-drug interactions at hOAT1 could be expected only in case of high plasma concentrations of ETV caused by overdosing or intoxication. Our finding support several clinical studies that reported that ETV and adefovir or tenofovir can be administrated together without the risk of pharmacokinetic interaction (Bifano et al., 2007; Jiménez-Pérez et al., 2010).

CNT carriers are responsible for the high-affinity concentrative reabsorption of natural nucleosides and their analogs in the kidney (Ritzel et al., 2001; Rodríguez-Mulero et al., 2005); CNT2 mediates the uptake of antiviral purine derivatives such as cladribine, fludarabine, and clofarabine (Lang et al., 2001; King et al., 2006; Cano-Soldado and Pastor-Anglada, 2012). Thus, we hypothesized the same effect for ETV and our aim was to clarify for the first time the interactions of ETV with nucleoside transporters hCNT2 and hCNT3. Our findings indicate that the interaction of ETV with these two CNTs expressed in the kidney (Mangravite et al., 2003; Klaassen and Aleksunes, 2010) could be significant in terms of the renal handling of ETV (**Figure 3**). Because the CNTs are located at the apical membrane of the renal tubular cells (Mangravite et al., 2003) they may mediate reabsorption of ETV from the urine. Although CNT2 and CNT3 mRNA expression in the human kidney is several times lower than that of hOAT1 (Nishimura and Naito, 2005), the different cellular location may lead to different exposure to the drug. If we consider that about 70% of ETV dose is excreted via the urine (Matthews, 2006), the reached concentration in the final urine following administration of 1 mg could be in case of 24 h-urinary volume of 1 L approximately 700 μg/L (2.5 μM). Such concentration would be relatively high in comparison with that in plasma but not reaching the found Km of ETV for hCNT2 and hCNT3. However, CNTs are located in the proximal tubules (Klaassen and Aleksunes, 2010) where drug concentration in the ultrafiltrate is close to that in the plasma. Since ETV concentration is rising along the proximal tubules continually due to obligatory fluid reabsorption it is difficult to assess if the effective transport concentrations might be reached *in vivo*. Because secretion of ETV is the predominant process in the kidney, the tested hCNT transporters probably do not play under standard situations a quantitatively important role in ETV renal transport. In contrast to ETV, none of the tested antivirals (adefovir, cidofovir, and tenofovir) interacted with hCNT2 or with hCNT3 in the transiently transfected MDCK II cells (**Figure 5**). Therefore, no interactions involving CNT2 or CNT3 between ETV and these antivirals can be expected.

The accumulation of drugs in the cells mediated by influx transporters may be responsible for the resulting cytoxicity. In Ho et al. (2000) documented a critical role of OAT1 in the cytotoxic effect of adefovir and cidofovir *in vitro* using a cellular model. Adefovir, cidofovir, and tenofovir have been proved to possess a significant nephrotoxic potential in treated patients (Izzedine et al., 2005). Since OAT1 is typically abundantly expressed in the kidney (Klaassen and Aleksunes, 2010), the toxic potency of these drugs may be at least partly related to this transporter. In experiments aimed at OAT1-mediated cytotoxicity we found a markedly lower affinity of ETV to OAT1 and a lower cytotoxic effect of ETV in the cells transfected with hOAT1 in comparison with the above-mentioned nephrotoxic antivirals. Therefore, a lower risk of cellular accumulation may be expected for ETV than with adefovir, tenofovir, and cidofovir in tissues with a high expression of OAT1. In the publication by Ho et al. (2000), the authors determined Km of adefovir 23.8 μM and Km of cidofovir 58.0 μM in CHO cells stably expressing hOAT1. After 5 days of incubation with the tested antiviral drugs, the authors performed MTT cytotoxicity assay showing higher cytotoxic potential of adefovir than cidofovir. In our experiments in HeLa cells transiently transfected with hOAT1 we found *K*_m_ of ETV 316.5 μM suggesting lower uptake of ETV by hOAT1 and thus lower potential for cytotoxicity in comparison with adefovir and cidofovir. In accordance with this, our results from the study on hOAT1-mediated cytotoxicity measured by MTS method in HeLa cells after 24 h of incubation indicate that the least cytotoxic antiviral drug was ETV followed by cidofovir and adefovir. The differences between the cytotoxicity of antiviral drugs presented in these two papers may stem from different incubation times of antiviral drugs with the hOAT1 expressing cells (in Ho’s paper 5 days, in our study 1 day), type of cells and the method of transfection, but the general order of the cytotoxicity of the tested antivirals seems to be the same in both studies. Because of the found significant interaction of ETV with hCNTs, we also tested potential transporter-mediated cytotoxicity related to the hCNT2 or hCNT3. Similar results were obtained because any sign of the transporter-mediated toxicity was not detected. Therefore, the cytotoxicity of ETV dependent on these two transporters seems to be also not probable. Our results on lacking cytotoxicity of ETV are in accordance with previously published data that demonstrated, that ETV is a safe and generally well tolerated drug used in patients with chronic HBV infection (Liaw et al., 2011). Nevertheless, prolonged administration of a nucleoside analog to any patient may enhance the risk of toxic reactions, especially in patients with impaired renal or hepatic function (Scott and Keating, 2009).

## Conclusion

Based on our data we suppose that the risk of ETV nephrotoxic effect caused by accumulation via the tested transporters is unlikely under standard therapeutic conditions. The presented study confirms *in vitro* a potency of high ETV concentrations to compete with adefovir, tenofovir, and cidofovir at the transporter hOAT1. We also for the first time demonstrate that ETV is an inhibitor and substrate of CNTs. However, the found ETV interactions with the studied transporters are likely to be manifested in the kidney only under the special conditions associated with high levels of ETV, such as absolute or relative overdosing.

## Author Contributions

Participated in research design: FT, JM, ZJ, JP. Conducted experiments: JM, MV, LN, PB, LH. Performed data analysis: FT, PP, ZJ, JP, LH. Wrote or contributed to the writing of the manuscript: FT, JM, PP, ZJ, JP.

## Conflict of Interest Statement

The authors declare that the research was conducted in the absence of any commercial or financial relationships that could be construed as a potential conflict of interest.
